# Supplementary material for: CNTN5-/+or EHMT2-/+human iPSC-derived neurons from individuals with autism develop hyperactive neuronal networks
Source: eLife. 2019 Feb 12;8:e40092. doi: 10.7554/eLife.40092 (PMC6372285; doi:10.7554/eLife.40092)
Supplement: Transparent reporting form [file elife-40092-transrepform.docx]

***eLife’s* transparent reporting form**

We encourage authors to provide detailed information *within their submission* to facilitate the interpretation and replication of experiments. Authors can upload supporting documentation to indicate the use of appropriate reporting guidelines for health-related research (see [EQUATOR Network](http://www.equator-network.org/%20)), life science research (see the [BioSharing Information Resource](https://biosharing.org/)), or the [ARRIVE guidelines](http://www.plosbiology.org/article/info:doi/10.1371/journal.pbio.1000412) for reporting work involving animal research. Where applicable, authors should refer to any relevant reporting standards documents in this form.

If you have any questions, please consult our Journal Policies and/or contact us: [editorial@elifesciences.org](mailto:editorial@elifesciences.org).

**Sample-size estimation**

- You should state whether an appropriate sample size was computed when the study was being designed
- You should state the statistical method of sample size computation and any required assumptions
- If no explicit power analysis was used, you should describe how you decided what sample (replicate) size (number) to use

Please outline where this information can be found within the submission (e.g., sections or figure legends), or explain why this information doesn’t apply to your submission:

The second paragraph of the “Discussion” section explains the sample size consideration, as follows:

***“To increase the modeling scalability of complex genetic disorders such as ASD while optimizing statistical power, several parameters require careful consideration. Given substantial variation in reprogramming and neuronal differentiation efficiencies, sample size is important to control. It was recently proposed that inter-individual variation, i.e., the number of probands with similar genetic variants, is more important to consider than intra-individual variation, i.e., the number of iPSC clones derived from the same individual (Hoffman et al., 2018). Aiming at multi-variant phenotyping, we tested one or two probands per deficient gene, however, we were able to create an isogenic pair in a different genetic background for the two highly relevant genes, i.e., CNTN5 (Lionel et al., 2011, Mercati et al., 2017, van Daalen et al., 2011) and EHMT2 (Deimling et al., 2017, Kleefstra et al., 2005, Zylicz et al., 2015), thereby controlling inter-individual variation. We derived two independent iPSC clones per participants to regulate intra-individual variation.”***

Moreover, we elected to use MEA as a screening tool because we had previously evaluated the sensitivity of our protocol. For example, MEA was used to confirm a hypoactive neuronal phenotype, previously detected using patch-clamp recordings, in 4 out of 5 KO lines, in which 6-8 MEA wells were recorded per line per experiment, in mostly 4 independent experiments (Deneault *et al*., Stem Cell Reports, 2018). With such a high level of sensitivity, plus testing usually 2 different lines per individual, we assumed that aiming at 6 technical and 3 biological replicates per line would support enough sensitivity for our screen.

**Replicates**

- You should report how often each experiment was performed
- You should include a definition of biological versus technical replication
- The data obtained should be provided and sufficient information should be provided to indicate the number of independent biological and/or technical replicates
- If you encountered any outliers, you should describe how these were handled
- Criteria for exclusion/inclusion of data should be clearly stated
- High-throughput sequence data should be uploaded before submission, with a private link for reviewers provided (these are available from both GEO and ArrayExpress)

Please outline where this information can be found within the submission (e.g., sections or figure legends), or explain why this information doesn’t apply to your submission:

The numbers of independent biological and technical replicates are summarized in Supplementary file 3, and this table is referred in each concerned figure legend.

No data exclusion was done, as stated in the second paragraph of the “Multi-electrode array (MEA)” section of “Materials and Methods”: ***“No non-active well was excluded in the analysis.”***

**Statistical reporting**

- Statistical analysis methods should be described and justified
- Raw data should be presented in figures whenever informative to do so (typically when N per group is less than 10)
- For each experiment, you should identify the statistical tests used, exact values of N, definitions of center, methods of multiple test correction, and dispersion and precision measures (e.g., mean, median, SD, SEM, confidence intervals; and, for the major substantive results, a measure of effect size (e.g., Pearson's r, Cohen's d)
- Report exact p-values wherever possible alongside the summary statistics and 95% confidence intervals. These should be reported for all key questions and not only when the p-value is less than 0.05.

Please outline where this information can be found within the submission (e.g., sections or figure legends), or explain why this information doesn’t apply to your submission:

Each concerned figure legend contains the appropriate statistical analysis method information, with values of n, multiple test correction, and p values.

(For large datasets, or papers with a very large number of statistical tests, you may upload a single table file with tests, Ns, etc., with reference to sections in the manuscript.)

**Group allocation**

- Indicate how samples were allocated into experimental groups (in the case of clinical studies, please specify allocation to treatment method); if randomization was used, please also state if restricted randomization was applied
- Indicate if masking was used during group allocation, data collection and/or data analysis

Please outline where this information can be found within the submission (e.g., sections or figure legends), or explain why this information doesn’t apply to your submission:

This information is indicated in the first paragraph of the “Result” section:

## Selection and Collection of Tissue Samples for Reprogramming

Participants were enrolled in the Autism Speaks MSSNG whole-genome sequencing (WGS) project (Yuen et al., 2017). All ASD and related control-participants were initially consented for WGS and upon return of genetic results, then consented for the iPSC study, using approved protocols through the Research Ethics Board at the Hospital for Sick Children (see Materials and Methods section for details) (Hoang et al., 2018b). Some families were also examined by whole exome sequencing. The study took place over a 5-year period and used incrementally developing ASD gene lists from the following papers (Jiang et al., 2013, Marshall et al., 2008, Tammimies et al., 2015, Yuen et al., 2015) (**Table 1**). These primarily considered data from the Autism Speaks MSSNG project, the Autism Sequencing Consortium (De Rubeis et al., 2014), and the Simons Foundation Autism Research Initiative (SFARI) gene list (discussion below). A diversity of different ASD-risk variants was targeted ranging in size from single nucleotide variants (SNV) to an 823 kb CNV (**Figure 1** and **Table 1**; corresponding genomic coordinates in **Supplementary File 1**). Typically, one ASD-affected and one sex-matched unaffected member (control) per family were included (**Figure 1**). In total, 14 ASD-affected and 11 controls participated, of which 21 were males and 4 were females (**Figure 1** and **Table 1**). Cells from either skin fibroblasts or CD34+ blood cells were collected for reprogramming into iPSCs (**Figure 2A** and **Table 1**).

**Additional data files (“source data”)**

- We encourage you to upload relevant additional data files, such as numerical data that are represented as a graph in a figure, or as a summary table
- Where provided, these should be in the most useful format, and they can be uploaded as “Source data” files linked to a main figure or table
- Include model definition files including the full list of parameters used
- Include code used for data analysis (e.g., R, MatLab)
- Avoid stating that data files are “available upon request”

Please indicate the figures or tables for which source data files have been provided:

Figure 3; Figure 3-figure supplement 1; Figure 4; Figure 5; Figure 6; Figure 6-figure supplement 2
